# Regulation of cAMP accumulation and activity by distinct phosphodiesterase subtypes in INS-1 cells and human pancreatic β-cells

**DOI:** 10.1371/journal.pone.0215188

**Published:** 2019-08-23

**Authors:** Evan P. S. Pratt, Kyle E. Harvey, Amy E. Salyer, Gregory H. Hockerman

**Affiliations:** 1 Department of Medicinal Chemistry and Molecular Pharmacology, Purdue University, West Lafayette, IN, United States of America; 2 Purdue University Interdisciplinary Life Science Program, Purdue University, West Lafayette, IN, United States of America; Broad Institute, UNITED STATES

## Abstract

Pancreatic β-cells express multiple phosphodiesterase (PDE) subtypes, but the specific roles for each in β-cell function, particularly in humans, is not clear. We evaluated the cellular role of PDE1, PDE3, and PDE4 activity in the rat insulinoma cell line INS-1 and in primary human β-cells using subtype-selective PDE inhibitors. Using a genetically encoded, FRET-based cAMP sensor, we found that the PDE1 inhibitor 8MM-IBMX, elevated cAMP levels in the absence of glucose to a greater extent than either the PDE3 inhibitor cilostamide or the PDE4 inhibitor rolipram. In 18 mM glucose, PDE1 inhibition elevated cAMP levels to a greater extent than PDE3 inhibition in INS-1 cells, while PDE4 inhibition was without effect. Inhibition of PDE1 or PDE4, but not PDE3, potentiated glucose-stimulated insulin secretion in INS-1 cells. PDE1 inhibition, but not PDE3 or PDE4 inhibition, reduced palmitate-induced caspase-3/7 activation, and enhanced CREB phosphorylation in INS-1 cells. In human β-cells, only PDE3 or PDE4 inhibition increased cAMP levels in 1.7 mM glucose, but PDE1, PDE3, or PDE4 inhibition potentiated cAMP levels in 16.7 mM glucose. Inhibition of PDE1 or PDE4 increased cAMP levels to a greater extent in 16.7 mM glucose than in 1.7 mM glucose in human β-cells. In contrast, elevation of cAMP levels by PDE3 inhibition was not different at these glucose concentrations. PDE1 inhibition also potentiated insulin secretion from human islets, suggesting that the role of PDE1 may be conserved between INS-1 cells and human pancreatic β-cells. Our results suggest that inhibition of PDE1 may be a useful strategy to potentiate glucose-stimulated insulin secretion, and to protect β-cells from the toxic effects of excess fatty acids.

## Introduction

Pancreatic β-cells secrete the blood glucose-lowering hormone insulin to maintain glucose homeostasis in the body [1]. Pancreatic β-cell dysfunction and cell death underlies the development of type 2 diabetes [2]. At the cellular level, glucose-stimulated insulin secretion (GSIS) is driven by Ca^2+^ influx through the L-type voltage-gated Ca^2+^ channels (L-VGCC) Ca_v_1.2 and Ca_v_1.3 [3], and release of Ca^2+^ from the endoplasmic reticulum (ER) [4]. GSIS is further regulated by the second messenger 3',5'-cyclic adenosine monophosphate (cAMP), which is generated by the enzyme adenylyl cyclase (AC) [5]. The transmembrane ACs (tmAC) AC1, AC5 and AC8 and soluble AC (sAC) are primarily responsible for cAMP production in β-cells [6–8]. In addition to enhancing GSIS, cAMP promotes pancreatic β-cell mass through increased replication [9] and decreased apoptosis [10]. Both glucose [8, 11, 12] and incretin hormones [13], such as glucagon-like peptide-1 (GLP-1), are capable of stimulating cAMP production and subsequent activation of the cAMP effector proteins Protein Kinase A (PKA) and Exchange Protein Directly Activated by cAMP (Epac) [14]. PKA and Epac regulate insulin secretion through proximal effects on the machinery involved in exocytosis at the plasma membrane [15–17] and distal effects on ER Ca^2+^ release channels [18, 19]. cAMP signaling is compartmentalized to microdomains within the cell, including near sites of ER Ca^2+^ release, by phosphodiesterase enzymes (PDE), which degrade cAMP to 5’-AMP.

PDE1, PDE3, PDE4, and PDE8 are widely-regarded as the primary PDE subtypes responsible for regulating cytosolic cAMP levels and GSIS in rodent β-cell lines, and rodent and human islets [20]. PDE1 is the only subtype that is regulated by Ca^2+^/Calmodulin [21, 22] and is predicted to serve a critical role in pancreatic β-cells where Ca^2+^ dynamics and signaling are prominent [23]. Indeed, the subtype-selective PDE1 inhibitor 8-methoxymethyl-3-isobutyl-1-methylxanthine (8MM-IBMX) elevated resting and glucose-stimulated cAMP levels and enhanced GSIS in βTC3 cells [23], MIN6 cells, primary mouse β-cells [24] and intact islets from rat and human [25]. Yet, PDE3 appears to be the primary PDE in mouse, rat, and human islets, and the pancreatic β-cell lines INS-1 and MIN6. PDE3 overexpression led to reduced glucose-stimulated cAMP production, impaired GSIS [26, 27] and glucose intolerance [28], whereas PDE3 inhibition [24, 25, 29] or PDE3 knockdown [30, 31] elevated resting and glucose-stimulated cAMP levels and GSIS [32]. It has also been suggested that leptin and insulin-like growth factor 1 attenuate insulin secretion through PDE3B activation [33, 34]. Subtype-selective inhibitors and knockdown of PDE4 or PDE8 also elevated resting cAMP levels in INS-1 cells [30, 32], βTC3 cells [23], and intact rat and human islets [25, 29], suggesting that PDE4 and PDE8 may too be required. PDE8 knockdown enhanced GSIS in INS-1 cells [30, 32] and MIN6 cells [24], which directly correlates with PDE8-mediated regulation of resting cAMP levels. PDE4 inhibition, on the other hand, enhanced GSIS in clonal pancreatic β-cell lines [30, 32] but not in primary islets [25, 29], revealing a disconnect between clonal and primary β-cells. Furthermore, PDE4 activity was elevated in MIN6 cells and primary mouse β-cells following stimulation with glucose, as compared with resting conditions [24]. The paucity of information regarding PDE activity in human β-cells and the disparate results in various rodent models have hampered the development of selective PDE inhibition as a strategy for treatment of type 2 diabetes.

Using subtype-selective inhibitors of PDE1, PDE3, PDE4, and PDE8, we performed a side-by-side comparison of the role of each subtype in INS-1 cells and for the first time, in single human pancreatic β-cells. We used a state-of-the-art fluorescent sensor of cAMP to evaluate PDE-mediated regulation of cAMP under resting conditions and during glucose stimulation. The latter allowed us to directly compare our findings with the ability of each PDE inhibitor to potentiate GSIS. Furthermore, we revealed that PDE inhibition may potentiate GSIS in INS-1 cells independently of changes in cytosolic Ca^2+^. Finally, we show that PDE inhibition not only enhanced GSIS but also promoted INS-1 cell survival in the face of stress.

## Materials and methods

### Chemicals and reagents

D-glucose was purchased from Mallinckrodt Chemicals (Dublin, Ireland). IBMX (3-Isobutyl-1-methyl-1H-purine-2,6(3H,7H)-dione) was purchased from Sigma-Aldrich (St. Louis, MO). 8MM-IBMX (3-Isobutyl-8-(methoxymethyl)-1-methyl-1H-purine-2,6(3H,7H)-dione) was purchased from Santa Cruz Biotechnology (Dallas, TX). Cilostamide (N-cyclohexyl-N-methyl-4-(1,2- dihydro-2-oxo-6-quinolyloxy) butyramide), rolipram (4-[3-(cyclopentyloxy)-4-methoxyphenyl]-2-pyrrolidinone), and PF-04671536 (5-Methyl-3-[[(2R)-4-(2-thiazolylmethyl)-2-morpholinyl]methyl]-3H-1,2,3-triazolol[4,5]pyrimidin-7-amine) were purchased from Tocris Bioscience (Bristol, UK). PF-04957325 (3-[[(2R)-4-(1,3-thiazol-2-ylmethyl)morpholin-2-yl]methyl]-5-(trifluoromethyl)triazolo[4,5-d]pyrimidin-7-amine) was purchased from MedChemExpress (Monmouth Junction, NJ). Unless otherwise indicated, all other reagents were purchased from Sigma-Aldrich.

### INS-1 cell culture

INS-1 cells (ATCC, Manassas, VA) were cultured in complete RPMI-1640 media containing 11 mM glucose, 10 mM HEPES, 10% fetal bovine serum (FBS), 11 mg/mL sodium pyruvate, 10,000 units/mL penicillin, 10,000 μg/mL streptomycin, and 50 μM β-mercaptoethanol at 37°C and 5% CO_2_ [35]. In some experiments, INS-1 cells were serum-deprived overnight using minimal RPMI-1640 media containing 2.5 mM glucose, 10 mM HEPES, 11 mg/mL sodium pyruvate, 10,000 units/mL penicillin, 10,000 μg/mL streptomycin and 50 μM β-mercaptoethanol supplemented with 0.1% fatty acid-free bovine serum albumin (BSA).

### Intracellular Ca^2+^ assay

INS-1 cells were plated in 96-well black-walled plates (Corning Life Sciences, Corning, NY) and incubated for at least 24h at 37°C and 5% CO_2_. Cells were washed once with 200 μL Phosphate Buffered Saline (PBS) and incubated in 100 μL Krebs-Ringer HEPES Buffer (KRBH) containing 5 μM Fura2-AM (Molecular Probes, Eugene, OR) for 1h at room temperature. KRBH contained 134 mM NaCl, 3.5 mM KCl, 1.2 mM KH_2_PO_4_, 0.5 mM MgSO_4_, 1.5 mM CaCl_2_, 5 mM NaHCO_3_ and 10 mM HEPES and was supplemented with 0.05% fatty-acid free BSA (pH 7.4). Following this 1h incubation, KRBH containing Fura2-AM was removed, and the cells were washed once with 200 μL KRBH. Cells were pretreated for 30 min with 100 μL KRBH containing the working concentration of inhibitors or KRBH alone. Intracellular Ca^2+^ assays were performed using a Synergy 4 Multi-Mode Plate Reader (BioTek Instruments, Winooski, VT). Fura2 fluorescence was measured using a 508/20 nm band-pass emission filter and 340/11 nm or 380/20 nm band-pass excitation filters. For acute application of caffeine or PDE inhibitors, a 15 sec baseline was recorded prior to injection of 100 μL of a 2X concentration of stimulant, and fluorescence was recorded for an additional 2 min. Fluorescence was collected from both channels every 0.7 sec. For long-term stimulation with PDE Inhibitors in combination with glucose, a 5 min baseline was recorded prior to injection of 100 μL of a 2X concentration of stimulant, and fluorescence was recorded for an additional 1h. Fluorescence was collected from both channels every 0.7 sec. To assess changes in cytosolic Ca^2+^, the fluorescent signal collected using the 340 nm excitation filter was divided by the fluorescent signal collected using the 380 nm excitation filter (340/380 nm) for each well over the entire time-course. The average ratio during the first min was subtracted from the entire time-lapse to yield a baseline of zero. Each treatment was performed in quadruplicate, and the average 340/380 nm ± SE was determined for the time-course. At least three independent experiments were performed for each treatment, and the average AUC ± SE was determined.

### Insulin secretion

INS-1 cells were plated in 24-well plates (Corning Life Sciences, Corning, NY) and incubated for at least 24h at 37°C and 5% CO_2_. Cells were washed once with PBS and incubated with 500 μL KRBH alone or containing the working concentration of inhibitors for 30 min at 37°C and 5% CO_2_. Following pretreatment, 500 μL KRBH containing a 2X concentration of glucose or glucose + PDE inhibitors along with the working concentration of the same inhibitors used in the pretreatment was added to each well and incubated for an additional 1h at 37°C and 5% CO_2_. Following this incubation, the supernatant (1 mL) was transferred from each well to 1.5 mL Eppendorf tubes and stored at 20°C until assayed. Secreted insulin was measured using the High-Range Insulin ELISA kit (ALPCO, Salem, NH), according to the manufacturer’s instructions. After supernatant removal, cells from each well were immediately lysed in 200 μL Cell lysis buffer (20 mM Na_2_HPO_4_ + 150 mM NaCl + 0.1% Triton X-100, pH 7.4) + freshly added protease inhibitors (800 nM aprotinin, 50 μM leupeptin, 1 μg/mL pepstatin, 1 mM benzamidine, 1 mM 4-(2-Aminoethyl) benzenesulfonylfluoride,10 μg/mL calpain inhibitor I and 10 μg/mL calpain inhibitor II). Cell lysis buffer was transferred from each well to 1.5 mL tubes, placed on ice for 20 min and centrifuged at 14,000 x g for 10 min. The supernatant was transferred to fresh 1.5 mL tubes and stored at -20°C until assayed. Cellular protein was measured using the Pierce BCA Protein Assay Kit (Thermo Fisher Scientific, Waltham, MA), according to the manufacturer’s instructions. Each sample was assayed in duplicate. Secreted insulin was normalized to protein content, both of which were measured using the PowerWave Plate Reader (BioTek Instruments, Winooski, VT). A calibration curve was generated using standards provided in each kit, and the amount of secreted insulin and protein content were determined. The amount of insulin secreted (ng) was divided by protein content (mg) to yield an insulin/protein ratio for each well. Each treatment was performed in triplicate, and the average insulin/protein ratio was calculated among like wells. The average insulin/protein ratio of each treatment was normalized to the untreated condition to yield percent basal. This value was again normalized to the glucose treatment to yield percent glucose. At least three independent experiments were performed for each treatment, and the average percent glucose ± SE was determined.

### CREB phosphorylation assay

INS-1 cells were plated in 96-well black-walled plates and incubated for at least 24h at 37°C and 5% CO_2_. Cells were incubated for an additional 24h in minimal RPMI media. Cells were washed twice with PBS and incubated for 2h in KRBH at 37°C and 5% CO_2_. The preincubation buffer was removed and replaced with KRBH alone or containing the working concentration of inhibitors for 30 min at 37°C and 5% CO_2_. Cells were treated for 10 min with KRBH alone or KRBH containing the working concentration of PDE inhibitors. Following this incubation, cells were fixed with 4% formaldehyde and stored at 4°C until assayed. Total CREB and pCREB were measured using the Human/Mouse/Rat Phospho-CREB (S133) Cell-based ELISA (R&D Systems, Minneapolis, MN), according to the manufacturer’s instructions. Using a Synergy 4 Multi-Mode Plate Reader (BioTek Instruments, Winooski, VT), total CREB was measured at 450 nm with excitation at 360 nm, and pCREB was measured at 600 nm with excitation at 540 nm. The fluorescent signal of pCREB was divided by the fluorescent signal of total CREB to yield a pCREB/total CREB ratio for each well. Each treatment was performed in duplicate, and the average pCREB/total CREB was calculated between these two wells. The average pCREB/total CREB among background wells (no primary antibody) was subtracted from the values obtained from other wells. The average pCREB/total CREB ratio of each treatment was normalized to the untreated condition to yield percent basal. In some cases, the basal value was subtracted and the resulting values were normalized to the glucose treatment to yield percent glucose. At least three independent experiments were performed for each treatment, and the average percent basal or percent glucose ± SE was calculated.

### Caspase-3/7 Glo assay

INS-1 cells were plated in 96-well white-walled plates (Corning Life Sciences, Corning, NY) and incubated for at least 24h at 37°C and 5% CO_2_. INS-1 cells were treated with a 3:1 molar ratio of palmitate to BSA, with and without the working concentration of PDE inhibitors, or BSA alone (untreated control). To prepare the stock solution of palmitate, sodium palmitate (100 mM) (Santa Cruz Biotechnology, Dallas, TX) was dissolved in 50% ethanol by incubating at 55°C for 10–15 min with frequent vortexing. As an untreated control, 50% ethanol that did not contain palmitate was also prepared. A 1:200 dilution (50 μL) of palmitate (500 μM final) or control solution (0.25% ethanol final) was added to 10 mL minimal RPMI media containing 1% fatty acid-free BSA. Tubes containing palmitate + BSA or BSA alone were incubated in a 37°C water bath for 30 min to promote conjugation. INS-1 cells were treated for 12h at 37°C and 5% CO_2_. Following incubation, treatments were removed and replaced with 50 μL of fresh media. 50 μL of Caspase-3/7 Glo Reagent (ProMega, Madison, WI) was added to each well to yield a 100 μL total volume. As a background control, 50 μL RPMI-1640 + 50 μL Caspase-3/7 Glo Reagent were added to several wells that did not contain INS-1 cells. The plate was shaken at 500 rpm for 30 sec and incubated in the dark at room temperature for 2h. Luminescence was recorded using a Synergy 4 Multi-Mode Plate Reader (BioTek Instruments, Winooski, VT). A 1s integration time was used in all experiments; however, the sensitivity was changed to achieve an average luminescence recording of 500 in the background wells. Each treatment was performed in triplicate, and the average luminescence signal was calculated among these wells. The average luminescent signal of each treatment was normalized to the untreated condition to yield percent basal. This value was again normalized to palmitate to yield percent palmitate. At least three independent experiments were performed for each treatment, and the average percent palmitate ± SE was determined.

### Human pancreatic islets

Human islets were acquired from the Integrated Islet Distribution Program (City of Hope National Medical Center, Duarte, CA). The average purity and viability of the human islet preparations used in these studies was 92% and 94%, respectively. All the donors were classified as nondiabetics, and the cause of death was reported as stroke (4 donors), head trauma (4 donors), anoxia (2 donors), or CNS tumor (1 donor). The average age was 40 years old (range 28–62), the average weight was 225.3 pounds (range 170–296) and the average body mass index was 33.0 (range 23.7–49.9). Upon receipt, human islets were centrifuged at 200 x g for 5 min and re-suspended in RPMI-1640 media containing 11 mM glucose, 10 mM HEPES, 10% FBS, 10,000 units/mL penicillin and 10,000 μg/mL streptomycin (human islet media). Islets were plated in 24-well plates (30–40 islets/well) for FRET-based imaging experiments or ultra-low attachment 60 mm culture dishes (Corning Life Sciences, Corning, NY) for single islet insulin secretion assay. Islets were incubated for at least 24h at 37°C and 5% CO_2_ for recovery.

### Dissociation and transfection of human pancreatic islets

Human islets were dissociated from one well of a 24-well plate (30–40 islets). Islets were transferred to a 1.5 mL Eppendorf tube and centrifuged at 200 x g for 5 min. The media was carefully removed and replaced with 1 mL warm Versene (Thermo Fisher Scientific, Waltham, MA). The islets were re-suspended by pipetting up and down 5X, and centrifuged at 200 x g for 5 min. The Versene was carefully removed and replaced with 1 mL warm Accutase (Thermo Fisher Scientific, Waltham, MA). The islets were re-suspended by pipetting up and down 5X and placed on a rocker at room temperature for 5 min for constant agitation. Following agitation, the islets were briefly placed in a 37°C water bath and re-suspended by pipetting up and down 5X. The 1.5 mL tube was placed on the rocker at room temperature for an additional 5 min. Following agitation, the islets were briefly warmed in a 37°C water bath and re-suspended for a final time by pipetting up and down 5X. The dissociated islets were pelleted at 200 x g for 5 min, and the Accutase solution was carefully removed and replaced with 100 μL human islet media. The islets were resuspended and transferred to the center of a 40 mm coverslip coated with poly-D-lysine placed in a 60 mm dish. The dissociated islet cells were allowed to settle for 10–15 min prior to transfection. 500 ng Epac-S^H187^ + 1 μL P3000 + 1 μL Lipofectamine 3000 (Life Technologies, Grand Island, NY) was used for transfection. The DNA + P3000 and lipofectamine 3000 were added to separate 1.5 mL tubes, each containing 10 μL OPTI-MEM. The contents of both tubes were combined to yield a total volume of 20 μL, and the DNA and lipofectamine were allowed to complex at room temperature for 5 min. Following 4-6h incubation at 37°C and 5% CO_2_, fresh human islet media was carefully added to the 60 mm dish as not to disturb the dissociated islets. Dissociated cells were imaged at 48h post-transfection.

### Single human islet insulin secretion assay

Using a dissection microscope, single human islets (50–100 μm in diameter) were transferred from an ultra-low attachment 60 mm dish to 96-well V-bottom clear-walled plate (Corning Life Sciences, Corning, NY) containing 100 μL human islet media and incubated for 48h at 37°C and 5% CO_2_. Following this incubation, the cells were washed once with 50 μL warm KRBH + 1.7 mM glucose and incubated with 100 μL warm KRBH + 1.7 mM glucose for 30 min at 37°C and 5% CO_2_. The pretreatment was removed and replaced with 100 μL warm KRBH + 1.7 mM glucose (1.7G) or KRBH + 16.7 mM glucose (16.7G) with and without the working concentration of PDE inhibitors, and incubated for an additional 1h at 37°C and 5% CO_2_. The supernatants containing secreted insulin were transferred to fresh wells on the same 96-well V-bottom plate. Single human islets were lysed using 100 μL RIPA Buffer (50 mM Tris + 150 mM NaCl + 1% Triton X-100 + 0.5% Sodium Deoxycholate + 0.1% SDS) + freshly added protease inhibitors. The 96-well V-bottom plate containing cell lysate and secreted insulin was wrapped in parafilm and stored at 20°C until assayed. Secreted insulin and insulin content were diluted 1:10 and 1:100, respectively, to fall in the dynamic range of the kit, and each sample was assayed in duplicate using the Human Insulin Chemiluminescence ELISA (ALPCO, Salem, NH), according to the manufacturer’s instructions. Within 5–15 min of adding the chemiluminescent substrate, luminescence was detected using the Synergy 4 Multi-Mode Plate Reader using a 1s integration time and detector sensitivity of 150. A calibration curve was generated using standards provided in the kit, and the amount of secreted insulin and insulin content were determined. The resulting values were corrected for dilution, and the amount of secreted insulin was divided by total insulin (secreted insulin + insulin content) to yield a secreted insulin/total insulin percentage for each well. Each treatment was performed in triplicate, and the average % secreted/total insulin ± SE was calculated.

### FRET-based cAMP imaging

**Fig 1:**
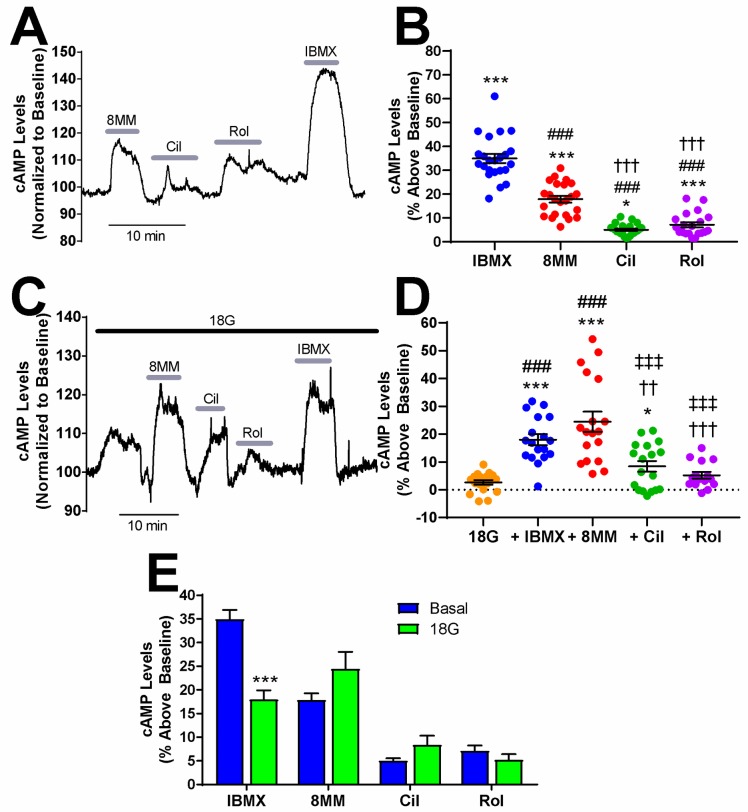
PDE1 regulates basal cAMP levels and glucose-stimulated cAMP in INS-1 cells. A) Subtype-selective PDE inhibitors and the pan PDE inhibitor IBMX raise basal cAMP levels in INS-1 cells. The Epac-S^H187^ mTurquoise2/FRET ratio is directly proportional to the cAMP level. Data shown is a representative experiment from a single cell. **B)** The percent increase in cAMP levels above baseline elicited by PDE inhibitors in INS-1 cells under basal conditions. IBMX (100 μM), 8MM-IBMX (100 μM), cilostamide (1 μM) and rolipram (10 μM) significantly elevate cAMP levels above baseline. Further, the percent increase elicited by each of the subtype-selective inhibitors is significantly less than that of IBMX. Last, 8MM-IBMX caused a significantly greater elevation in cAMP levels than either cilostamide or rolipram (***, P < 0.001, *, P < 0.05 compared to baseline; ###, P < 0.001 compared to IBMX; †††, P < 0.001 compared to 8MM-IBMX; One-way ANOVA, Tukey post-hoc test). Data shown are mean ± SE from 23 cells taken from four independent experiments (three outliers removed from rolipram analysis).**C)** Subtype-selective PDE inhibitors and the pan PDE inhibitor IBMX raise cAMP levels in the presence of 18 mM glucose in INS-1 cells. Data shown is a representative experiment from a single cell. **D)** The percent increase in cAMP levels above baseline elicited by PDE inhibitors in INS-1 cells in the presence of 18 mM glucose. IBMX, 8MM-IBMX and cilostamide significantly elevate cAMP levels above baseline, whereas only IBMX and 8MM-IBMX are significantly greater than glucose. IBMX and 8MM-IBMX caused a significantly greater elevation in cAMP levels than either cilostamide or rolipram (***, P < 0.001, *, P < 0.05 compared to baseline; ###, P < 0.001 compared to 18G; †††, P < 0.001, ††, P < 0.01 compared to IBMX; ‡‡‡, P < 0.001 compared to 8MM-IBMX; One-way ANOVA, Tukey post-hoc test). Data shown are mean ± SE from 18 cells taken from four independent experiment (three outliers removed from rolipram analysis). **E)** Comparison of cAMP accumulation stimulated by PDE inhibitors in the absence and presence of 18 mM glucose. IBMX stimulated a greater increase in cAMP levels in the absence of glucose (basal), compared to 18 mM glucose (***, *P* < 0.001; Student’s unpaired t-test).

**Fig 2:**
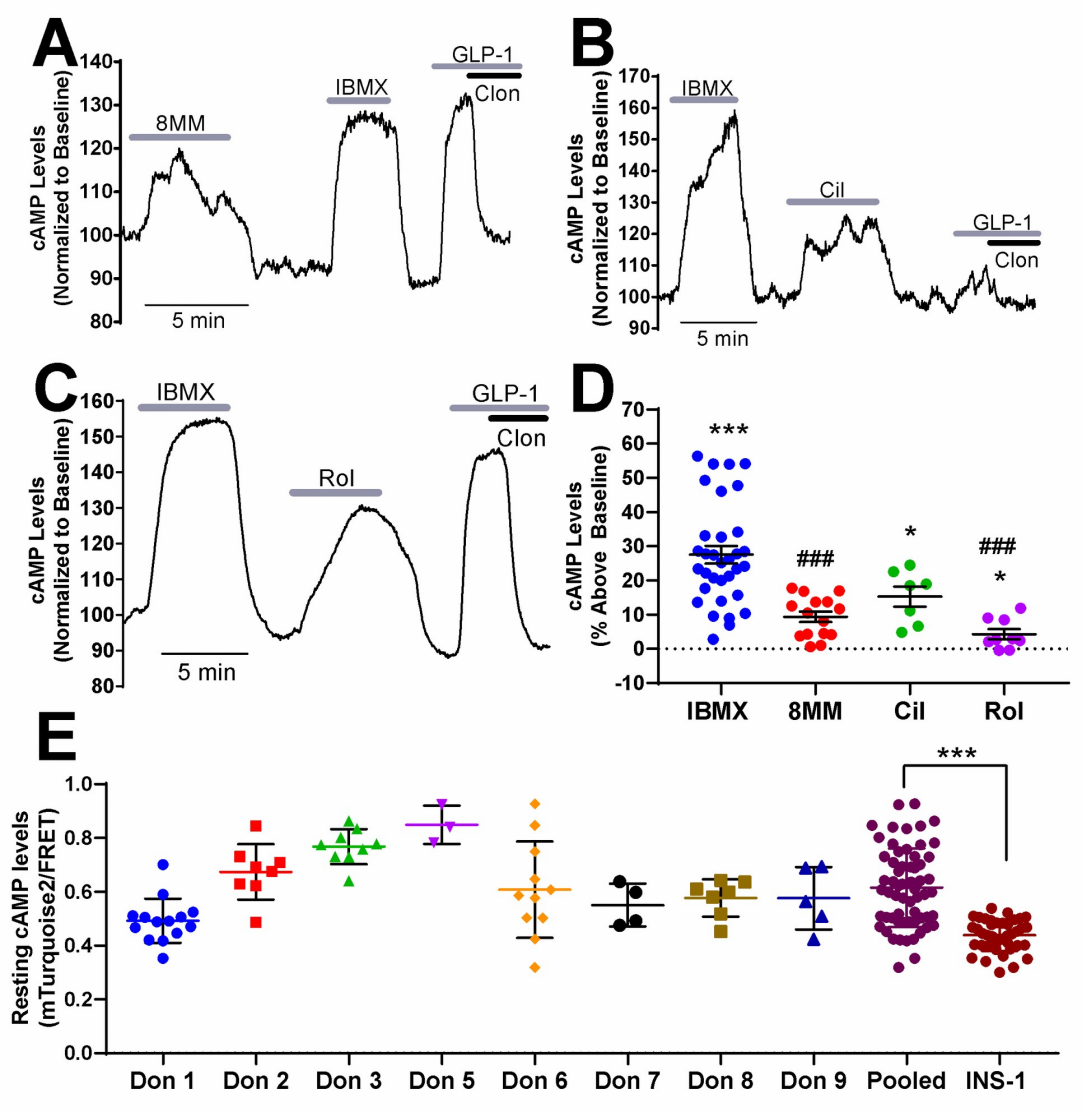
Regulation of basal cAMP levels by PDEs in human pancreatic β-cells. A-C) Subtype-selective PDE inhibitors and IBMX raise cAMP levels in pancreatic β-cells dissociated from human islets under basal conditions (1.7 mM glucose). For each experiment, IBMX (100 μM) and either 8MM-IBMX (100 μM) (A), cilostamide (1 μM) (B) or rolipram (10 μM) (C) were perfused onto the cell. Pancreatic β-cells were identified at the end of each experiment by addition of GLP-1 (50 nM) with and without the α2-receptor agonist clonidine (1 μM). Data shown are representative experiments from single human pancreatic β-cells. D) The percent increase in cAMP levels above baseline elicited by PDE inhibitors in human pancreatic β-cells under basal conditions. IBMX, cilostamide and rolipram significantly elevate cAMP levels above baseline. The percent increase in cAMP levels stimulated by 8MM-IBMX and rolipram is significantly less than that of IBMX (***, P < 0.001, *, P < 0.05 compared to baseline; ###, P < 0.001 compared to IBMX; One-way ANOVA, Tukey post-hoc test). Data shown are average ± SE from 15 cells (8MM-IBMX), 7 cells (cilostamide), 9 cells (rolipram) and 33 cells (IBMX), collected from four human islet preparations. One outlier was removed for IBMX and three outliers were removed for rolipram. E) Resting cAMP levels (mTurquoise2/FRET) in β-cells from eight different human donors. Comparison of pooled data from human β-cells (61 total) to resting cAMP levels in INS-1 cells (41 total) revealed a significantly greater resting cAMP level in human β-cells (***, *P* < 0.001; Student’s unpaired t-test). Individual data points are shown for each cell measured. Lines represent means ± SE.

**Fig 3:**
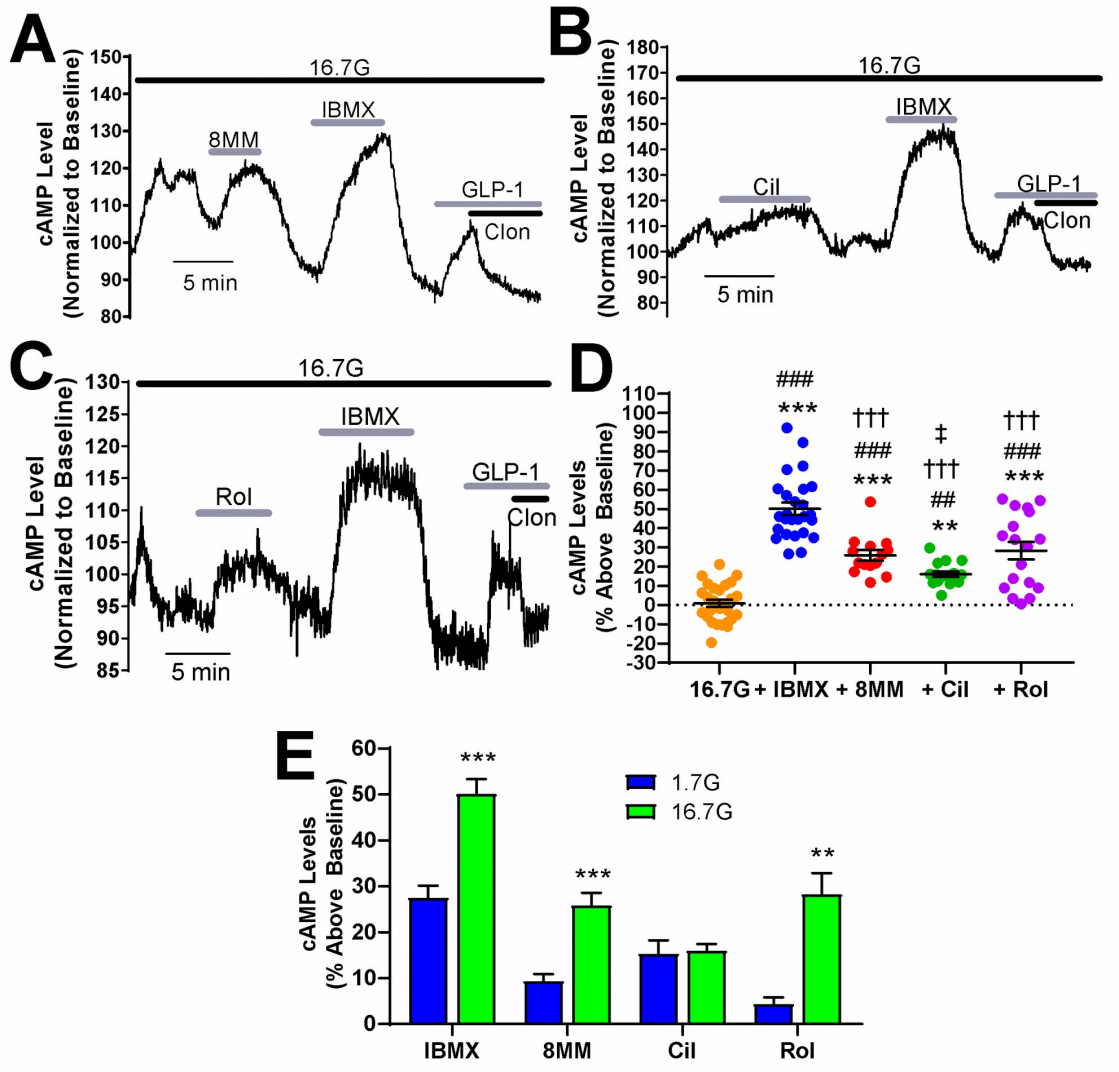
Regulation of glucose-stimulated cAMP by PDEs in human pancreatic β-cells. A-C) Subtype-selective PDE inhibitors and IBMX raise cAMP levels in pancreatic β-cells dissociated from human islets under high glucose (16.7 mM) stimulation conditions. For each experiment, IBMX (100 μM) and either 8MM-IBMX (100 μM) (A), cilostamide (1 μM) (B) or rolipram (10 μM) (C) were perfused onto the cell. Pancreatic β-cells were identified using GLP-1 and clonidine as described for Fig 2. Data shown are representative experiments from single human pancreatic β-cells. D) The percent increase in cAMP levels above baseline elicited by PDE inhibitors in human pancreatic β-cells in the presence of 16.7 mM glucose. IBMX, 8MM-IBMX, cilostamide, and rolipram all significantly elevate cAMP levels above baseline and 18G. Furthermore, 8MM-IBMX, cilostamide and rolipram result in significantly less cAMP accumulation compared with IBMX. Last, cilostamide stimulates significantly less cAMP accumulation than rolipram (***, *P* < 0.001 compared to baseline; ###, *P* < 0.001, ##, P < 0.01 compared to 18G; †††, *P* < 0.001 compared to IBMX; ‡, P < 0.05 compared to rolipram; One-way ANOVA with Tukey post-hoc test). Data are shown as mean ± SE from 26 cells (IBMX), 14 cells (8MM-IBMX), 17 cells (cilostamide), and 18 cells (rolipram), collected from four human islet preparations. One outlier was removed for IBMX treatment. E) Comparison of cAMP accumulation stimulated by PDE inhibitors in 1.7 mM (basal) or 16.7 mM glucose. IBMX, 8MM-IBMX, and rolipram stimulated a greater increase in cAMP levels in 16.7 mM glucose, compared to 1.7 mM glucose (***, *P* < 0.001, **, *P* < 0.01; Student’s unpaired t-test).

To measure cAMP accumulation in the presence of PDE inhibitors, INS-1 cells were transfected with 1 μg Epac-S^H187^ DNA using 2.5 μL Lipofectamine 2000 (Life Technologies, Grand Island, NY), according to the manufacturer’s instructions. Following 4-6h incubation at 37°C and 5% CO_2_, the cells were split into 60 mm dishes containing 40 mm glass coverslips coated with poly-D-lysine and imaged at 48h post-transfection. INS-1 cells and dissociated human islet cells were imaged using a Nikon A1 Confocal and Perfect Focus Ti-E Inverted Microscope equipped with an Apo TIRF 60x Oil DIC N2 (NA 1.49) objective lens. 40 mm coverslips were assembled into a RC-31 imaging chamber (Warner instruments, Hamden, CT) and attached to a six-channel perfusion mini-valve system (VC-6M). The donor signal of Epac-S^H187^ (mTurquoise2) was collected using the following confocal parameters: 457 nm laser line of a Multi-Argon laser (457/476/488/514 nm), a 400-457/514 nm primary dichroic mirror, a 520 nm long-pass dichroic mirror and a 485/35 nm band-pass emission filter. The FRET signal of Epac-S^H187^ was collected using the following confocal parameters: 457 nm laser line of a Multi-Argon laser (457/476/488/514 nm), a 400-457/514 nm primary dichroic mirror, a 565 nm long-pass dichroic mirror and a 538/33 nm band-pass emission filter. Confocal images of donor and FRET signal were collected sequentially every 2 sec using the following parameters: 512 x 512 total pixels, 4.8 μs pixel dwell, pinhole size of 1.2 AU and varied zoom factor, laser power and detector gain. To assess changes in cAMP levels in INS-1 cells and human β-cells, we calculated the ratio of Epac-S^H187^ mTurquoise2 signal divided by the FRET signal (mTurquoise2/FRET) using NIS Elements 4.0 image analysis software. In all experiments, a resting baseline was collected for 1 min prior to the addition of PDE inhibitors (Fig 1A and 1B and Fig 2) or glucose (Fig 1C and 1D and Fig 3), and representative traces were normalized to this baseline. For experiments performed under resting conditions in INS-1 cells (Fig 1A and 1B) and human β-cells (Fig 2), cAMP imaging was performed exclusively in KRBH containing no glucose or 1.7 mM glucose, respectively. After establishing a stable baseline, PDE inhibitors were sequentially applied to INS-1 cells and human β-cells, with a KRBH wash separating the addition of each drug. For experiments performed in the presence of glucose in INS-1 cells (Fig 1C and 1D) and human β-cells (Fig 3), a resting baseline was recorded in KRBH containing no glucose or 1.7 mM glucose, respectively, prior to the addition KRBH containing 18 mM glucose (INS-1 cells) or 16.7 mM glucose (human β-cells). Cells were exposed to KRBH + glucose for 5–10 min followed by sequential addition of PDE inhibitors (suspended in KRBH + glucose) to INS-1 cells and human β-cells. A wash step with KRBH + glucose separated the addition of each drug. In experiments with INS-1 cells, all four PDE inhibitors were applied in the following order: 8MM-IBMX, cilostamide, rolipram and IBMX. Experiments with human β-cells were performed using two approaches: (1) as described for INS-1 cells in which all four PDE inhibitors were applied in the same order or (2) only one subtype-selective PDE inhibitor (8MM-IBMX, cilostamide, rolipram) was applied followed by addition of the pan PDE inhibitor IBMX. Furthermore, GLP-1 and clonidine were used to identify human β-cells at the end of these experiments. cAMP levels increased following the addition of KRBH + PDE inhibitors (Fig 1A and 1B and Fig 2), KRBH + glucose (Fig 1C and 1D and Fig 3), and KRBH + glucose + PDE inhibitors (Fig 1C and 1D and Fig 3), eventually achieving a stable donor/FRET ratio or plateau in most cells. Once cAMP levels stabilized, treatments were washed off using KRBH (Fig 1A and 1B and Fig 2) or KRBH + glucose (Fig 1C and 1D and Fig 3). Once cAMP levels stabilized near resting levels, the next treatment was added. To quantify the effect of KRBH + PDE inhibitors, KRBH + glucose and KRBH + glucose + PDE inhibitors on cAMP levels, we calculated the average donor/FRET ratio achieved by each treatment and subtracted resting cAMP levels to yield a percent change in cAMP levels above baseline. While the first treatment in each experiment was normalized to the initial resting baseline, subsequent treatments were normalized to the donor/FRET ratio observed immediately before treatment addition to account for changes in resting cAMP levels observed during wash steps. At least three independent experiments were performed for each treatment in INS-1 cells and human β-cells, and the average percent increase in cAMP levels ± SE was determined.

### Data analysis

Data were analyzed using GraphPad Prism 7. Data are represented as the average ± SE. Statistical analysis was performed using one-way ANOVA with the Tukey post-hoc test (multiple comparisons). The student’s unpaired t-test was used for pair-wise comparisons. *P* < 0.05 was considered significant. Outliers were identified using the ROUT method (Q = 1), and deleted from analysis as indicated in the legends to Figs 1, 2 and 3.

## Results

### PDE-mediated regulation of resting and glucose-stimulated cAMP levels in INS-1 cells

The role of PDE1, PDE3, PDE4 and PDE8 in regulating both resting and glucose-stimulated cAMP levels in pancreatic β-cells is poorly understood. We used the Epac1-based, high-affinity, high-dynamic range FRET sensor Epac-S^H187^ to evaluate the effect of subtype-selective PDE inhibitors on cAMP levels in INS-1 cells with real-time resolution [36]. We transiently transfected INS-1 cells with Epac-S^H187^ and imaged cells by confocal microscopy at 48 hours post-transfection in KRBH without glucose. Application of the pan PDE inhibitor IBMX (100 μM) resulted in a robust increase in cAMP levels above baseline levels (34.99 ± 1.94%) that recovered to baseline upon drug removal (Fig 1A). We sequentially applied the subtype-selective inhibitors 8MM-IBMX, cilostamide, and rolipram to test the role of the IBMX-sensitive PDE1, PDE3, and PDE4, respectively (Fig 1A). The concentrations of PDE inhibitors used in this study were 5–20 times the reported IC_50_ values [37–39] and were comparable with those used in a similar study performed in MIN6 cells [24]. Application of 100 μM 8MM-IBMX (17.87 ± 1.42%), 1 μM cilostamide (5.04 ± 0.5%), and 10 μM rolipram (7.15 ± 1.1%) significantly elevated cAMP levels above baseline (Fig 1B). Notably, 8MM-IBMX had a significantly greater effect on resting cAMP levels than either cilostamide and rolipram. Thus, PDE1, PDE3, PDE4 regulate resting cAMP levels in INS-1 cells with a rank order of PDE1 > PDE3 = PDE4. Since PDE8 is not inhibited by IBMX [40], we examined the effect of the PDE8-selective inhibitor PF-04957325 [41] on resting cAMP levels in INS-1 cells. We found that 100 nM PF-04957325 (IC50 < 1 nM for both PDE8A and 8B) had no effect on cAMP levels under basal conditions (S1 Fig). Additionally, the PDE8-selective inhibitor PF-04671536 (IC_50_ < 2 nM for both PDE8A and 8B)(42), didn’t increase basal cAMP levels in INS-1 cells at concentrations up to 5 μM (S1 Fig).

PDE1 activity is upregulated by Ca^2+^/Calmodulin [21, 22], while PDE3 and PDE4 activities are upregulated by PKA-mediated phosphorylation [42]. Glucose elevates Ca^2+^ and cAMP levels in pancreatic β-cells [11]; therefore, it’s plausible that the PDE profile is altered in INS-1 cells following glucose stimulation. To test this, we stimulated INS-1 cells expressing Epac-S^H187^ with 18 mM glucose and sequentially applied the PDE inhibitors. Fig 1C shows initial application of glucose (18 mM) in some cells resulted in a small but detectable rise in cytosolic cAMP levels that dropped back to baseline levels or began to oscillate, as previously reported [11]. However, the average increase in cAMP levels (2.64 ± 0.83%) upon stepping glucose from 0 to 18 mM was not significantly different from resting levels. IBMX and 8MM-IBMX significantly elevated cAMP levels compared to those measured in 0 or 18 mM glucose (Fig 1D). Similar to our findings under resting conditions, 100 μM IBMX (18 ± 1.93%), 100 μM 8MM-IBMX (24.45 ± 3.61%), and 1 μM cilostamide (8.39 ± 1.92%) significantly elevated cAMP levels above baseline levels in the presence of glucose. In contrast, 10 μM rolipram (5.22 ± 1.21%) was without effect (Fig 1D). Furthermore, 8MM-IBMX had a significantly greater effect on glucose-stimulated cAMP levels compared with rolipram and cilostamide, suggesting that PDE1 is the primary regulator of cAMP levels under resting and glucose-stimulated conditions. In addition, we directly compared the effect of each PDE inhibitor under resting conditions with glucose stimulation. We found that for all of the PDE-selective inhibitors the extent of cAMP increase stimulated did not differ in the presence or absence of glucose (Fig 1E). Interestingly, the IBMX-induced rise in cAMP levels was significantly greater in the absence of glucose than in 18 mM glucose.

### PDE-mediated regulation of resting and glucose-stimulated cAMP levels in human β-cells

Given that the rat-derived INS-1 cell line may not reflect human β-cell physiology, we examined the roles of PDE1, PDE3, PDE4 and PDE8 in regulation of resting cAMP levels in isolated pancreatic β-cells dissociated from human islets. Previous reports have demonstrated that PDE1, PDE3 and PDE4 regulate cAMP levels in intact human islets; however, there is no evidence which suggests that this is specific to β-cells [25, 43]. Dissociated human islet cells were transiently transfected with Epac-S^H187^ and imaged at 48 hours post-transfection by confocal microscopy. To distinguish pancreatic β-cells from other islet cell types, we employed a previously established pharmacological approach [24]. Unlike other islet cell types, pancreatic β-cells express both the GLP-1 receptor and α2-adrenergic receptor, which when activated with their respective agonists, lead to AC activation and inhibition, respectively. At the end of each imaging experiment, we applied the GLP-1R agonist GLP-1 (50 nM) followed by co-administration of the α_2_-adrenergic receptor agonist clonidine (1 μM). If a cell exhibited a GLP-1-induced rise in cAMP levels that was dampened or abolished by application of clonidine, it was classified as a pancreatic β-cell (Fig 2A). We used this approach to measure cAMP levels in human pancreatic β-cells obtained from non-diabetic human donors (S1 Table) and found that, as in INS-1 cells, application of 100 μM IBMX in low glucose (1.7 mM) resulted in a robust increase in cAMP levels above baseline levels (27.53 ± 2.60%) (Fig 2). Further, 1 μM cilostamide (15.3 ± 2.94%) and 10 μM rolipram (4.33 ± 1.46%) significantly elevated resting cAMP levels, whereas 100 μM 8MM-IBMX (9.35 ± 1.53%) did not (Fig 2). This suggests that unlike INS-1 cells, PDE3 and PDE4, but not PDE1, regulates resting cAMP levels in human pancreatic β-cells. We also examined the role of PDE8 in regulating cAMP levels in human pancreatic β-cells. We used the PDE8 inhibitor PF-04671536 since it is reported to stimulate GSIS in human islets [44]. However, 5 μM PF-04671536 had no effect on basal cAMP levels in human pancreatic β-cells (S2 Fig). We also examined the resting cAMP levels in human β-cells from eight different donors and INS-1 cells by comparing the mTurquoise2/FRET ratio in 1.7 mM and 0 glucose, respectively, before the addition of any stimulus. We found that pooled data from human β-cells revealed a significantly higher resting cAMP level than INS-1 cells (Fig 2E).

To compare regulation of resting and glucose-stimulated cAMP levels by PDEs in human pancreatic β-cells, we examined the role of PDE1, PDE3, and PDE4 in regulation of cAMP levels during 16.7 mM glucose stimulation in human pancreatic β-cells from non-diabetic human donors (S1 Table). At the end of each experiment, pancreatic β-cells were again identified by the characteristic increase in cAMP concentration stimulated by GLP-1 which is rapidly reversed by clonidine (Fig 3A). Similar to INS-1 cells, glucose triggered an initial increase in cAMP levels, followed by cAMP oscillations in some cells. However, the average cAMP level was not significantly increased in human β-cells by stepping the glucose concentration from 1.7 mM (resting) to 16.7 mM (Fig 3D). We found that the pan PDE inhibitor IBMX (50.41 ± 3.20%), PDE1-selective inhibitor 8MM-IBMX (25.81 ± 2.75%), PDE3-selective inhibitor cilostamide (15.99 ± 1.4%), and PDE4-selective inhibitor rolipram (28.26 ± 4.59%) each significantly elevated cAMP levels above glucose-stimulated cAMP levels (Fig 3D). Rolipram stimulated significantly greater increases in cAMP levels in 16.7 mM glucose than did cilostamide. A direct comparison of PDE-mediated regulation of cAMP levels under resting (Fig 2) and stimulatory conditions (Fig 3) revealed that the IBMX-induced rise was significantly elevated in the presence of glucose (Fig 3E). Consistent with this, 8MM-IBMX and rolipram had a significantly greater effect in the presence of 16.7 mM glucose than in 1.7 mM glucose. However, the increase in cAMP stimulated by cilostamide was not different at these two glucose concentrations. Thus, PDE1 and PDE4 are upregulated following glucose stimulation, suggesting that the activity of these PDE subtypes is enhanced by elevated glucose concentrations in human β-cells. In the case of PDE1, activity is highly dependent on glucose stimulation in human pancreatic β-cells.

### PDE-mediated regulation of GSIS in INS-1 cells and human islets

**Fig 4:**
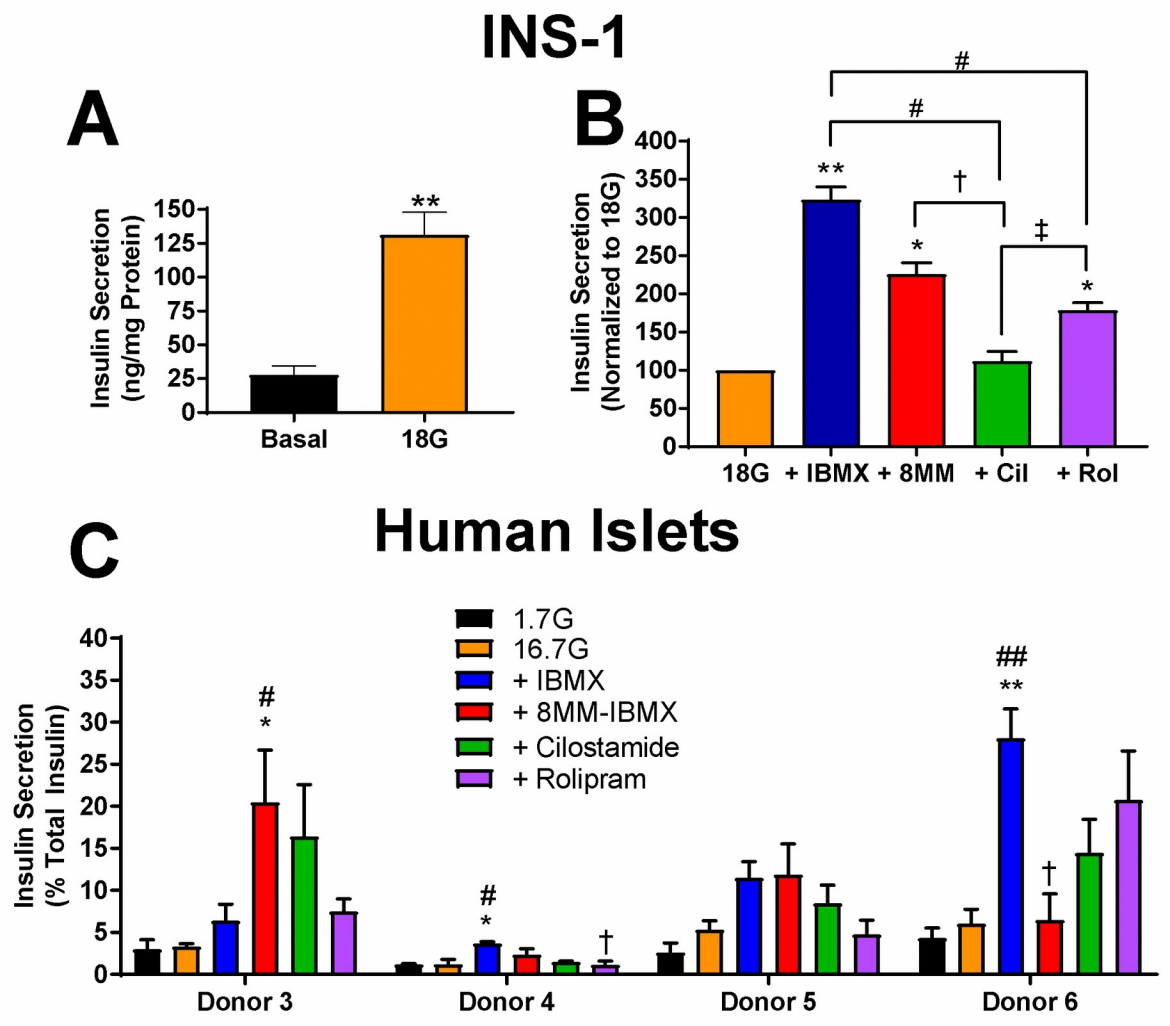
Effect of PDE inhibitors on GSIS in INS-1 cells and human pancreatic islets. A) Glucose (18 mM) significantly stimulates insulin secretion above basal levels in INS-1 cells (**, P < 0.01, compared to 0 glucose; Student’s unpaired t-test). Data shown are mean ± SE from four independent experiments. B) Subtype-selective PDE inhibitors and the pan PDE inhibitor IBMX potentiate insulin secretion stimulated with glucose (18 mM) in INS-1 cells. Each experiment was normalized to the glucose response. IBMX (100 μM), 8MM-IBMX (100 μM) and rolipram (10 μM) significantly stimulate insulin secretion, compared with glucose alone. Potentiation of GSIS with cilostamide (1 μM) or rolipram is significantly less than IBMX. Further, insulin secretion stimulated with cilostamide is significantly less than both 8MM-IBMX and rolipram (**, P < 0.01, *, P < 0.05 compared to 18G; #, P < 0.05 compared to IBMX; †, P < 0.05 compared to 8MM-IBMX; ‡, P < 0.05 compared to rolipram; One-way ANOVA, Tukey post-hoc test). Data shown are mean ± SE from four independent experiments. C) Subtype-selective PDE inhibitors and the pan PDE inhibitor IBMX potentiate insulin secretion stimulated with 16.7G in intact human islets isolated from four separate donors. In donor 3, 8MM-IBMX was significantly greater than 1.7G and 16.7G. In donor 4, IBMX was significantly greater than 1.7G and 16.7G, whereas rolipram was significantly less than IBMX. There are no significant differences among the treatment conditions for donor 5. Finally, in donor 6 we observed that IBMX had a significant effect above 1.7G and 16.7G, and 8MM-IBMX was less than IBMX (**, P < 0.01, *, P < 0.05 compared to 1.7G; ##, P < 0.01, #, P < 0.05 compared to 16.7G; †, P < 0.05 compared to IBMX; One-way ANOVA, Tukey post-hoc test). Data shown are mean ± SE from four human islet donors, in which each treatment was performed in triplicate.

Elevated cAMP concentrations can enhance GSIS [45]; therefore, we tested whether PDE-mediated regulation of glucose-stimulated cAMP levels in INS-1 cells (Fig 1) correlates with the ability of subtype-selective PDE inhibitors to potentiate GSIS. Here, we measured insulin secretion from INS-1 cells stimulated with 18 mM glucose in the absence or presence of PDE inhibitors. 18 mM glucose significantly stimulated insulin secretion from INS-1 cells compared to basal secretion (Fig 4A). Each experiment was normalized to insulin secretion in the presence of 18 mM glucose alone. Treatment with 100 μM IBMX resulted in greater than a three-fold increase (3.24 ± 0.17) in insulin secretion in INS-1 cells compared with glucose alone (Fig 4B). Treatment of INS-1 cells with 18 mM glucose + 100 μM 8MM-IBMX (2.25 ± 0.15) or 10 μM rolipram (1.79 ± 0.1) significantly potentiated insulin secretion compared with glucose alone, whereas 1 μM cilostamide (1.12 ± 0.13) did not (Fig 4B). Moreover, potentiation of GSIS by cilostamide was significantly less than that observed with IBMX, 8MM-IBMX, or rolipram. This suggests that PDE1 and PDE4, but not PDE3, regulate GSIS in INS-1 cells, consistent with our measurements of cAMP stimulation in Fig 1D, showing that PDE1 is the major subtype degrading cAMP in the presence of 18 mM glucose. However, the stimulation of GSIS from INS-1 cells by rolipram was surprising, given that it did not stimulate a significant increase in cAMP levels in 18 mM glucose. Lastly, we found that PDE8-selective inhibitor PF-04957325 (100 nM) did not significantly potentiate GSIS in INS-1 cells (S1 Fig). Thus, it appears that the major IBMX-insensitive PDE isoform found in pancreatic β-cells does not play a significant role in either regulation of basal cAMP levels or GSIS in INS-1 cells.

Given the distinct effects of PDE subtype inhibition on cAMP levels in human pancreatic β-cells, we evaluated whether inhibitors of PDE1, PDE3, and PDE4 potentiate GSIS in isolated human islets using a single-islet insulin secretion assay [46]. In short, islets obtained from non-diabetic human donors (S1 Table) were plated in a 96-well V-bottom plate, so that each well held a single islet. Human islets were incubated with PDE inhibitors at low glucose (1.7 mM) for 30 min prior to a 1h incubation with high glucose (16.7 mM) with and without PDE inhibitors. Insulin release was normalized to the insulin content of each islet. We found that 16.7 mM glucose stimulated a subtle increase in insulin secretion compared with 1.7 mM glucose in all four donors (Fig 4C), ranging from a 1.11-fold increase to 2.03-fold (S1 Table). However, in none of the donors did this apparent increase reach statistical significance. In contrast, the PDE inhibitors had more robust effects on GSIS in some donors. The pan PDE inhibitor IBMX trended toward potentiated GSIS in all four donors, and this reached statistical significance in two of the four donors (donors 4 and 6). The PDE1-selective inhibitor 8MM-IBMX enhanced GSIS in three donors (donors 3, 4 and 5), and this increase reached statistical significance in donor 3. The PDE3-selective inhibitor cilostamide trended toward potentiation of GSIS in three (donors 3, 5 and 6) and the PDE4-selective inhibitor rolipram trended toward significance in two (donors 3 and 6), but none of these increases reached statistical significance. Taken together, PDE1 appears to play a role in regulating GSIS in intact human islets, though there is clearly heterogeneity amongst individuals.

### PDE-mediated regulation of Ca^2+^ dynamics in INS-1 cells

**Fig 5:**
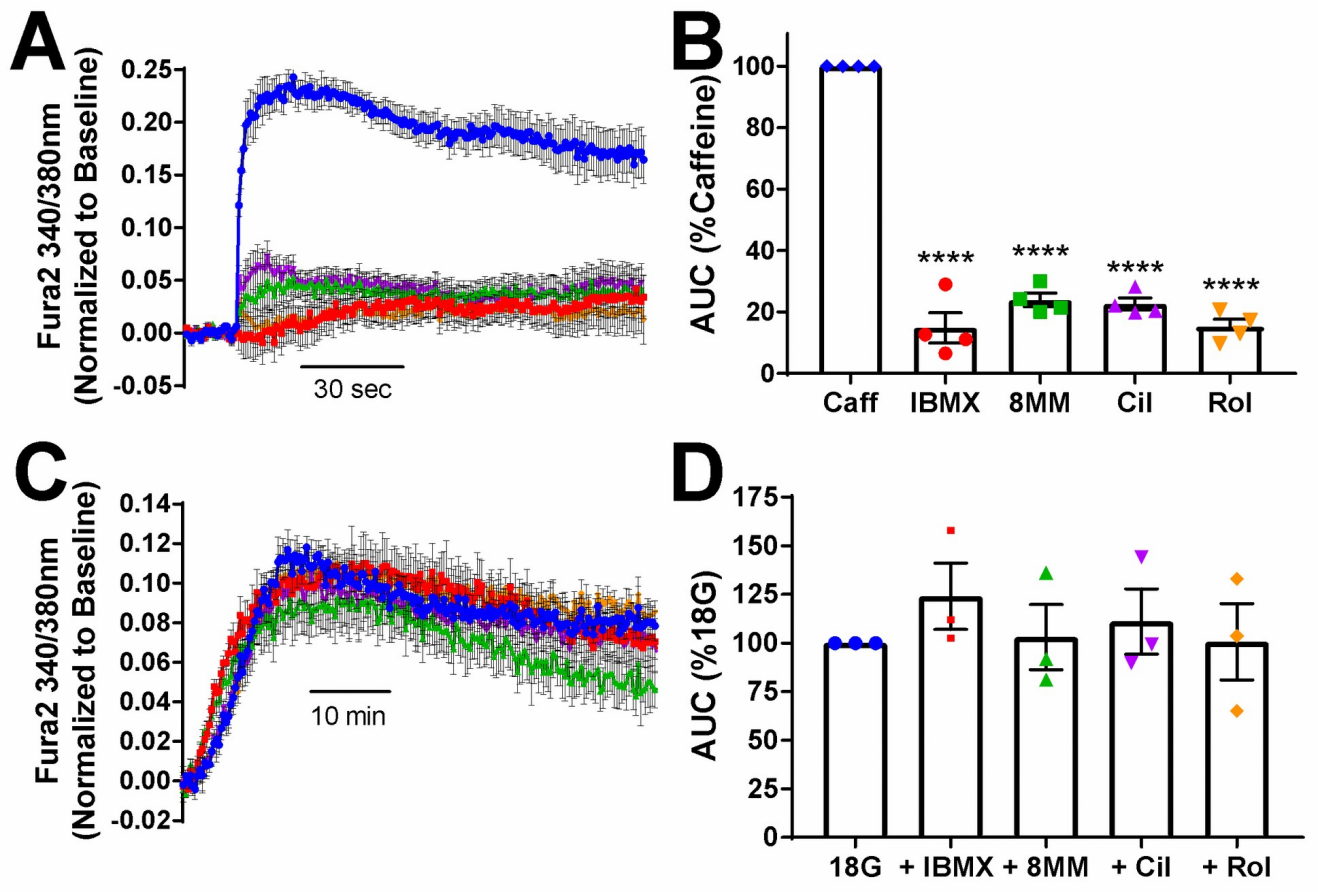
Effect of PDE inhibitors on intracellular Ca^2+^ dynamics in INS-1 cells. A) Caffeine robustly elevates intracellular Ca^2+^ in INS-1 cells when added acutely; however, the subtype-selective PDE inhibitors and the pan PDE inhibitor IBMX have little effect. Data shown are the mean ± SE from representative experiments performed in quadruplicate. B) AUC analysis of Ca^2+^ dynamics stimulated with caffeine (5 mM), 8MM-IBMX (100 μM), cilostamide (1 μM), rolipram (10 μM) or IBMX (100 μM). Each experiment was normalized to the response elicited by caffeine. The Ca^2+^ response elicited by each of the subtype-selective PDE inhibitors as well as IBMX was significantly less than that of caffeine (****, P < 0.0001 compared to caffeine; One-way ANOVA, Tukey post-hoc test). Data shown are average ± SE from four independent experiments. C) Glucose (18 mM) elevates intracellular Ca^2+^ levels in INS-1 cells but the subtype-selective PDE inhibitors and the pan PDE inhibitor IBMX have no additional effect. Data shown are the mean ± SE from representative experiments performed in quadruplicate. D) AUC analysis of Ca^2+^ dynamics stimulated with glucose (18 mM) alone and in the presence of either 8MM-IBMX (100 μM), cilostamide (1 μM), rolipram (10 μM) or IBMX (100 μM). Each experiment was normalized to the response elicited by 18G. There was no significant difference among the treatment conditions. Data shown are average ± SE from four independent experiments.

8MM-IBMX and rolipram potentiated GSIS in INS-1 cells (Fig 4B), suggesting that PDE1 and PDE4 are the major PDE subtypes that regulate GSIS in INS-1 cells. PDE-mediated regulation of GSIS presumably occurs following the rise in cytosolic cAMP levels (Fig 1) and subsequent activation of the cAMP effector proteins, PKA [47] and Epac [48]. However, both IBMX and 8MM-IBMX are derivatives of xanthine, as is caffeine, a strong activator of the ryanodine receptor (RyR) [49]. Therefore, we examined whether or not, like caffeine, IBMX and 8MM-IBMX, and potentially cilostamide and rolipram, enhanced GSIS by stimulating RyR-mediated Ca^2+^ release. Using the fluorescent Ca^2+^ sensor Fura2-acetoxymethyl (AM), we found that acute application of caffeine (5 mM) stimulated a rapid and robust increase in cytosolic Ca^2+^ (Fig 5A and 5B). In contrast, application of IBMX and each of the subtype-selective PDE inhibitors, at the concentrations used to stimulate cAMP accumulation, did not markedly elevate cytosolic Ca^2+^ levels in INS-1 cells. Quantification of the area under the curve (AUC) showed that all of the of the PDE inhibitors stimulated a significantly smaller increase in intracellular Ca^2+^ concentration compared to caffeine: 100 μM IBMX (14.86 ± 4.9%), 100 μM 8MM-IBMX (23.9 ± 2.23%), 1 μM cilostamide (22.67 ± 1.92%) or 10 μM rolipram (15.22 ± 2.41%). Thus, potentiation of GSIS by IBMX, 8MM-IBMX and rolipram in INS-1 cells is likely not due to direct activation of RyR. Alternatively, it’s possible that PDE-mediated enhancement of GSIS occurs via activation of PKA and Epac, which can enhance ER Ca^2+^ release [18, 19]. As expected, 18 mM glucose stimulated a rise in cytosolic Ca^2+^ that peaked approximately 10 min post-stimulation (Fig 5C). However, co-application of IBMX or any of the subtype-selective PDE inhibitors did not further increase intracellular Ca^2+^ levels in comparison with glucose alone (Fig 5D).

### PDE-mediated regulation of INS-1 cell survival

**Fig 6:**
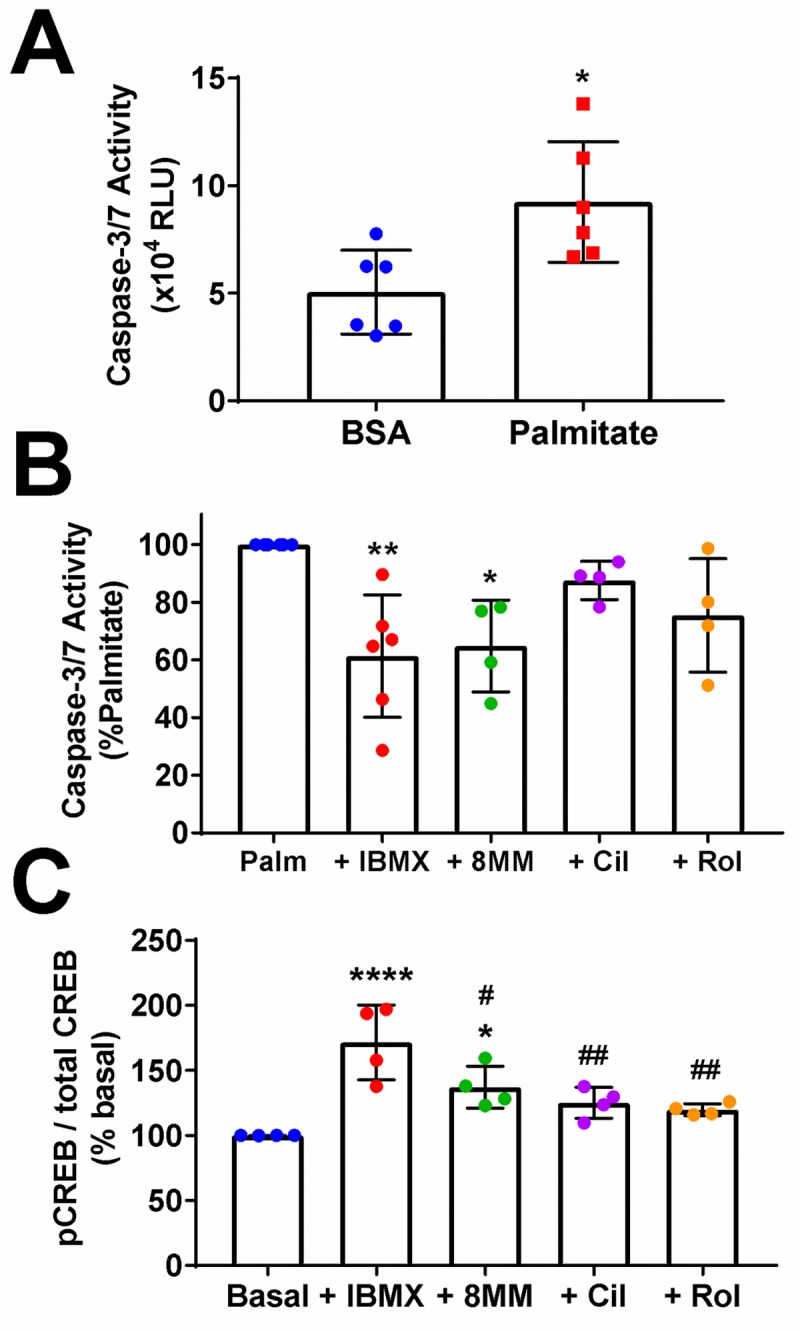
PDE1 inhibition reduces palmitate-induced apoptosis and stimulates CREB phosphorylation in INS-1 cells. A) Incubation of INS-1 cells with palmitate for 12h significantly elevates caspase-3/7 activation (*, P < 0.05 compared to BSA; Student’s unpaired t-test). Data shown are average ± SE from six independent experiments. B) The PDE1 inhibitor 8MM-IBMX (100 μM) and pan PDE inhibitor IBMX (100 μM) significantly decrease the level of caspase-3/7 activation induced by palmitate toxicity (**, P < 0.01, *, P < 0.05 compared to palmitate; One-way ANOVA, Tukey post-hoc test). Each experiment was normalized to the level of palmitate toxicity. Data shown are average ± SE from 4–6 independent experiments. C) The PDE1 inhibitor 8MM-IBMX (100 μM) and pan PDE inhibitor IBMX (100 μM) significantly stimulate CREB phosphorylation above basal levels (****, P < 0.0001, *, P < 0.05 compared to basal; ##, P < 0.01, #, P < 0.05 compared to IBMX; One-way ANOVA, Tukey post-hoc test). Data shown are average ± SE from four independent experiments.

cAMP not only enhances insulin secretion from pancreatic β-cells but also is an important regulator of β-cell mass, enhancing proliferation and cell survival [50]. Not surprisingly, cAMP signaling is impaired in diabetic pancreatic β-cells [51]. Given the robust effects of PDE inhibitors on cAMP levels (Fig 1), we tested whether raising cAMP levels using subtype-selective PDE inhibitors can rescue INS-1 cells from apoptosis caused by the saturated fatty acid palmitate, which is a widely-used model of lipotoxicity associated with type 2 diabetes [52]. We exposed INS-1 cells to palmitate (500 μM + 1% BSA: 3:1 molar ratio) [53] in the presence of PDE inhibitors and measured caspase-3/7 activation, an early marker of apoptosis, at 12 hours post-treatment [54]. Palmitate treatment significantly upregulated caspase-3/7 activity, compared with BSA alone (Fig 6A). Co-treatment of INS-1 cells with 100 μM IBMX (61.35 ± 8.65%) significantly decreased palmitate-induced caspase-3/7 activation (Fig 6B). Furthermore, we found that 100 μM 8MM-IBMX (64.81 ± 7.94%), but neither 1 μM cilostamide (87.54 ± 3.31%) nor 10 μM rolipram (75.45 ± 9.84%), significantly decreased palmitate-induced caspase 3/7 activation.

cAMP-response element binding protein (CREB) is a cAMP-dependent transcription factor that regulates gene transcription and pancreatic β-cell survival following PKA-dependent phosphorylation [10]. Thus, a potential mechanism by which IBMX and 8MM-IBMX may activate pro-survival pathways in INS-1 cells is through an elevation in cAMP levels (Fig 1) that ultimately leads to PKA-mediated phosphorylation of CREB. To determine whether PDE inhibition stimulates CREB phosphorylation, we treated INS-1 cells with IBMX and the subtype-selective PDE inhibitors for 10 min, lysed the cells and measured CREB phosphorylation at S133 (pCREB) and total CREB expression using a commercially available ELISA kit. We calculated the ratio of pCREB to total CREB and normalized each experiment to basal pCREB/total CREB. Using this approach, we found that 100 μM IBMX (171 ± 14.32%) and 100 μM 8MM-IBMX (137 ± 8.05%) significantly elevated pCREB/total CREB, compared with basal conditions (Fig 6C). In contrast, neither 1 μM cilostamide (125 ± 5.94% basal) nor 10 μM rolipram (120 ± 2.27% basal) had a significant effect. Taken together, the pro-survival effect of the PDE-selective inhibitor 8MM-IBMX could be the result of elevated cAMP levels (Fig 1) and subsequent activation of the cAMP-responsive transcription factor CREB.

## Discussion

In this study, we examined the role of PDE1, PDE3, PDE4, and PDE8 in regulating resting and glucose-stimulated cAMP levels and downstream signaling in INS-1 cells and primary human pancreatic β-cells. We found that PDE1, PDE3, and PDE4 but not PDE8, regulate resting cAMP levels, while PDE1 and PDE3, but not PDE4 regulate glucose-stimulated cAMP levels in INS-1 cells (Fig 1, S1 Fig). Inhibition of PDE1 had the greatest effect on cAMP levels under both conditions. In addition, PDE1 and PDE4 inhibition, but not PDE3 or PDE8 inhibition, enhanced GSIS in these cells (Fig 4B; S1 Fig). In contrast to INS-1 cells, we found that PDE3 and PDE4, but not PDE1 or PDE8, regulate resting cAMP levels in primary human β-cells (Fig 2, S2 Fig). However, in the presence of 16.7 mM glucose, PDE1 and PDE4 inhibition resulted in significantly higher cAMP levels compared with resting conditions, suggesting that these subtypes are upregulated by glucose stimulation in human β-cells (Fig 3). Consistent with this, among the subtype-selective inhibitors, only the PDE1-selective inhibitor 8MM-IBMX significantly enhanced insulin secretion from human islets obtained from any of the four donors used in this study (Fig 4C). Finally, we found that PDE1 inhibition reduced palmitate-induced caspase-3/7 activation (Fig 6B) and induced CREB phosphorylation (Fig 6C) in INS-1 cells. Taken together, our results suggest that PDE1-mediated regulation of cAMP levels is important for regulation of GSIS and pancreatic β-cell survival.

### PDE-mediated regulation of resting and glucose-stimulated cAMP levels in INS-1 cells and human pancreatic β-cells

We elected to study the role of PDE1, PDE3, PDE4, and PDE8 in PDE-mediated regulation of cAMP levels because they are among the most highly-expressed PDEs in pancreatic β-cell lines and islets from rat, mouse, and human [23, 30, 32, 43]. Subtype-selective inhibitors and siRNA-mediated knockdown have revealed an important role for PDE1, PDE3, PDE4, and PDE8 in regulation of resting cAMP levels and GSIS [23–25]. However, individual studies have focused on one or two PDE subtypes and have not performed a direct comparison of PDE1, PDE3, PDE4, and PDE8. The few studies that have examined PDE1, PDE3, PDE4, and PDE8 differed substantially in approach from the current study. For example, subtype-selective inhibitors of PDE1 and PDE4, but not PDE3, elevated cAMP content in homogenates of the mouse insulinoma β-cell line βTC3, suggesting that PDE1 and PDE4 are required [23]. However, studying the effect of PDE inhibitors on cAMP dynamics in living pancreatic β-cells in real-time, as we performed in this study, is more likely to reflect cAMP dynamics in vivo. A systematic comparison of PDE1, PDE3, PDE4, and PDE8 was performed in the mouse insulinoma cell line MIN6 and primary mouse pancreatic β-cells using a plasma membrane-localized fluorescent cAMP sensor and TIRF microscopy [24]. The authors concluded that PDE3 is the primary regulator of resting cAMP levels. However, PDE3 is commonly associated with cellular membranes, especially the plasma membrane [55, 56]. PDE1 and PDE4, on the other hand, are associated with cellular sub-compartments, including the ER [57]. Therefore, an approach that detects mainly sub-plasmalemmal cAMP may underestimate the contributions of PDE1 and PDE4. In the current study, we used a robust fluorescent cAMP sensor localized to the cytosol, Epac-S^H187^ [36], and found that under resting conditions, PDEs regulate resting cAMP levels in INS-1 cells with a rank order of PDE1 > PDE3 = PDE4 > PDE8 (Fig 1A and 1B, S1 Fig). In contrast, we found that in human pancreatic β-cells, PDE3 and PDE4, but not PDE1 or PDE8, regulate resting cAMP levels (Fig 2, S2 Fig). Two earlier studies have examined regulation of resting cAMP levels by PDE1, PDE3, and PDE4 in human islets [25, 43]. However, the authors measured cAMP in cell lysates obtained from intact islets, which included contributions from α- and δ-cells, in addition to β-cells [58]. Therefore, the present study, which specifically examines PDEs in isolated human pancreatic β-cells, is much less likely to be influenced by contributions from other cell types resident within the pancreatic islet.

While PDE-mediated regulation of resting cAMP levels has been extensively studied, regulation of cAMP levels by PDE1, PDE3, and PDE4 in the presence of glucose remains largely unexplored. This data is significant because it would be directly comparable with the effect of subtype-selective PDE inhibitors on GSIS. In this study, we found that stimulatory concentrations of glucose significantly altered the effect of IBMX in INS-1 cells (Fig 1E) and the effect of IBMX, 8MM-IBMX and rolipram in human β-cells (Fig 3E). The former might be explained by the localization of PDEs to different microdomains. PDE activity is governed by many factors including the local cAMP concentration within cAMP microdomains, which contain PDEs, ACs and the cAMP effectors PKA and Epac, and are anchored near target substrates throughout the cell, including at the plasma membrane [59], ER [60] and mitochondria [61] by A-Kinase Anchoring Protein (AKAP) [60, 62, 63]. ACs catalyze the production of cAMP, which along with PDEs, set the local concentration of cAMP [64]. Both transmembrane ACs [65] and soluble AC [5, 7, 66] are important for glucose-stimulated cAMP production and insulin secretion in β-cells. The tmACs AC1 [12] and AC8 [67, 68] directly couple glucose-stimulated Ca^2+^ influx with cAMP production and insulin secretion at the plasma membrane. In contrast, sAC is localized to the mitochondria [69], among other intracellular compartments, and is sensitive to not only changes in intracellular Ca^2+^ levels but HCO_3_^-^ and ATP as well, both of which become elevated during oxidative metabolism of glucose [70]. This suggests that unlike AC1 and AC8, sAC-mediated cAMP production may couple glucose metabolism with insulin secretion. There are likely to be several distinct cAMP microdomains present in β-cells which contain different components, such as PDE1 and tmAC or sAC. In pancreatic β-cells, evidence of a cAMP microdomain containing AKAP150, PKA and AC8 has been demonstrated [71–73]. In our studies, we found that the IBMX-stimulated rise in cAMP levels was significantly greater in the absence of glucose than in the presence of 18 mM glucose in INS-1 cells (Fig 1E); however, this was not the case for the subtype-selective inhibitors. Since the subtype-selective inhibitors roughly add up to IBMX under resting conditions (Fig 1B), this suggests that PDE1, PDE3 and PDE4 regulate distinct cAMP microdomains and are therefore coupled to different ACs pools (tmAC or sAC). However, in the presence of glucose, the distinct PDEs may transition to regulating a common cAMP microdomain in the cell. Indeed, stimulus-induced changes in the components of cAMP microdomains, including PDEs, have been reported previously [57, 74]. In future studies, it will be of interest to examine the components of cAMP microdomains in β-cells (e.g. coupling of PDEs with tmAC or sAC) and how glucose stimulation alters their composition and sub-cellular localization.

In contrast with our findings in INS-1 cells, the IBMX-stimulated rise in cAMP levels was significantly elevated following glucose stimulation of human β-cells (Fig 3E). PDE activity can be upregulated by protein kinase phosphorylation, such as PKA- and CamKII-mediated phosphorylation and upregulation of PDE4 activity [42, 75]. In addition, PDE1 activity is upregulated by Ca^2+^/Calmodulin [21, 22]. Since glucose elevates Ca^2+^ and cAMP levels, stimulating PKA activity [8, 11, 14], it’s possible that the increase in IBMX-sensitive PDE activity in human β-cells is due to glucose-mediated upregulation of PDE1 and PDE4 activities via regulation by Ca^2+^/Calmodulin and PKA, respectively. Indeed, 8MM-IBMX and rolipram treatment resulted in significantly greater cAMP levels in the presence of 16.7 mM glucose compared with 1.7 mM glucose, suggesting that PDE1 and PDE4 activity are upregulated by glucose stimulation, at least in human β-cells. This finding may also be explained in the context of the affinity of the different PDEs for cAMP. The resting cAMP concentration, which is dictated by tmACs in β-cells [5, 7], is estimated to be near 1 μM [76, 77], close to the K_m_ for cAMP of PDE1C and PDE4 isoforms, below the K_m_ for cAMP of PDE1A and 1B, but much higher than that of all PDE3 isoforms, which have K_m_ values for cAMP of between 20 and 150 nM [78]. Therefore, PDE3 activity is likely maximal under resting conditions, whereas the activities of PDE1 and PDE4 would be expected to increase in response to a glucose-stimulated elevation in cAMP levels.

### PDE-mediated regulation of Ca^2+^ dynamics and GSIS in INS-1 cells and human islets

We found that inhibition of PDE1 and PDE4 potentiated GSIS INS-1 cells (Fig 4B), while inhibition of PDE3 (Fig 4B) and PDE8 (S1 Fig) did not. Unfortunately, none of our experiments with human islets resulted in significant GSIS over basal secretion. However, in two donors (donors 4 and 6), IBMX increased insulin secretion over glucose alone, and in another donor (donor 3), inhibition of PDE1 significantly increased insulin secretion over glucose alone (Fig 4C). Inhibition of PDE1 trended toward potentiation of GSIS in two additional donors (donors 4&5). Our result in INS-1 cells is consistent with our finding that inhibition of PDE1 potentiates glucose-stimulated cAMP levels (Fig 1C and 1D). However, the ability of rolipram to stimulate GSIS in INS-1 cells despite no detectable effect on cAMP levels in 18 mM glucose suggests that a pool of cAMP not readily detected by the cytosolic cAMP sensor used in our experiments is capable of amplifying GSIS. Other studies on PDE-regulation of GSIS demonstrate that PDE3 is involved in regulating GSIS [26, 28–30, 79]. One exception to this is that the PDE1-selective inhibitor 8MM-IBMX was shown to potentiate GSIS in βTC3 cells [23], in agreement with our study. A direct comparison of GSIS regulation by PDE1, PDE3, PDE4, and PDE8 showed that knockdown of each subtype enhanced GSIS in INS-1 cells; however, there was no difference among them [32]. Another study found that PDE1 is an important regulator of GSIS in MIN6 cells, but also reported that PDE3 and PDE8 regulate GSIS as well [24]. In human islets PDE1, PDE3, and PDE4, together comprised the vast majority of total PDE activity, although other PDEs were detected by western blot [43]. Among the subtype-selective PDE inhibitors, inhibition of PDE1 resulted in the greatest amount of insulin secretion from human islets in most of the donors used in this study (Fig 4C). However, the fold-stimulation above glucose achieved by 8MM-IBMX and contribution of the remaining PDE subtypes, PDE3 and PDE4, greatly varied among experiments (Fig 4C). This variation may have arisen from biological differences among human donors, including age, cause of death or hemoglobin A1C, as previously reported using this same assay of insulin secretion [46].

The rise in cAMP levels stimulated by PDE inhibitors in INS-1 cells (Fig 1) and human pancreatic β-cells (Figs 2 and 3) likely resulted in activation of the cAMP effectors PKA and Epac, which are components of cAMP microdomains along with PDEs and ACs.[14] Within cAMP microdomains located at the plasma membrane, PKA and Epac enhance insulin secretion at the site of exocytosis through phosphorylation of exocytotic substrates [15] and direct interactions with scaffolding proteins [80], SNARE proteins [81] and ion channels [17], respectively. Within ER-localized cAMP microdomains, PKA and Epac can also enhance insulin secretion through more distal actions on ER Ca^2+^ channels IP_3_R and RyR. PKA directly phosphorylates the IP_3_R to stimulate ER Ca^2+^ release [82–84], whereas Epac is reported to regulate RyR-mediated Ca^2+^ release through a Rap1/PLCε/CamKII signaling pathway [18, 85]. Thus, we tested the possibility that PDE inhibition regulates [Ca^2+^]_in_ under various conditions, but did not detect any change in cytosolic Ca^2+^ levels with the subtype-selective PDE inhibitors or IBMX in the absence or presence of 18 mM glucose (Fig 5). Overall, this suggests that PDE1 inhibition in INS-1 cells and human islets enhances GSIS through more direct effects of cAMP.

### PDE inhibitors as a potential treatment for type 2 diabetes

As demonstrated by GLP-1 receptor stimulation, elevated cAMP levels not only enhance GSIS but can stimulate pancreatic β-cell proliferation [86] and cell survival [87]. Indeed, the pan PDE inhibitor IBMX has the ability to rescue pancreatic β-cells from cell death (Fig 6B) induced by the unsaturated fatty acid palmitate [88]. Palmitate exposure recapitulates key aspects of pancreatic β-cell dysfunction observed in diabetes, including disruption of glucose-stimulated cAMP oscillations, which is likely due to altered AC expression [51, 52]. We also showed that the PDE1-selective inhibitor 8MM-IBMX significantly reduces palmitate-induced caspase-3/7 activation in INS-1 cells, while the PDE3-selective inhibitor cilostamide and PDE4-selective inhibitor rolipram do not (Fig 6B). Consistent with our observation, PDE1A is strongly upregulated in INS-1 832/13 cells in response to 48 hr palmitate exposure [89].

Activation of the cAMP-response element binding protein (CREB) is associated with β-cell survival [10, 90]. Furthermore, IBMX has been shown to induce CREB phosphorylation via a PKA-dependent mechanism in other cell types [91]. In our study, we found that IBMX and 8MM-IBMX significantly stimulated CREB phosphorylation above resting levels, while cilostamide and rolipram did not (Fig 6C), suggesting that PDE1 inhibition may suppress pancreatic β-cell apoptosis via a CREB-dependent mechanism. Since GSIS and pancreatic β-cell mass are compromised in type 2 diabetics [2], it’s possible that PDE1 inhibitors could have clinical applications for treating this disease [92]. A recent study demonstrated that IBMX, the PDE3 inhibitors cilostamide and milrinone, the PDE4 inhibitor rolipram, and the PDE4/10 inhibitor dipyridamole stimulated rat pancreatic β-cell replication, and that the latter reduces serum glucose levels in humans [9]. However, the PDE1 inhibitor 8MM-IBMX did not significantly stimulate β-cell replication in this study, suggesting that PDE1 may regulate GSIS and pancreatic β-cell survival but not proliferation. It will be of interest to determine if specific PDE1 isoforms play distinct roles in the regulation of pancreatic β-cell function and survival.

## Supporting information

S1 Table Human islet donor characteristics. (DOCX)

S1 Fig PDE 8-selective inhibitors don’t potentiate cAMP levels or insulin secretion in INS-1 cells. (A) Application of 100 nM PF4957325 on INS-1 in KRBH with 0 glucose resulted in no increase in cAMP over baseline. Subsequent application of 100 μM IBMX resulted in increased cAMP ranging from 7–49% over baseline (n = 14 cells) (B) PF4957325 does not potentiate glucose-stimulated insulin secretion in INS-1 cells. Stimulation of INS-1 cells with 18 mM glucose resulted in a significant increase in insulin secretion over basal secretion. Co-stimulation with 100 nM PF4957325 failed to result in a significant increase in secretion compared to 18 mM glucose alone(n = 3). (C) PF04671536 does not increase intracellular cAMP in INS-1 cells. Application of increasing concentrations of PF04671536 to INS-1 cells in KRBH with 0 glucose failed to increase in cAMP over baseline. Application of 100 μM IBMX resulted in increases in cAMP ranging from 23–113% over baseline (n = 11 cells).
(TIF)

S2 Fig The PDE8-selective inhibitor PF04671536 does not increase basal cAMP levels in human pancreatic β-cells. Application of 100 nM PF04671536 to human β-cells in 1.7 mM glucose does not result in any increase in cAMP over baseline. Application of 100 μM IBMX increased cAMP ranging from 40–101%. GLP-1 and clonidine were used to identify β-cells (n = 4).
(TIF)
