# Supplementary material for: Regulation of cAMP accumulation and activity by distinct phosphodiesterase subtypes in INS-1 cells and human pancreatic β-cells
Source: PLoS One. 2019 Aug 23;14(8):e0215188. doi: 10.1371/journal.pone.0215188 (PMC6707593; doi:10.1371/journal.pone.0215188)
Supplement: S1 Table — (DOCX) [file pone.0215188.s001.docx]

| **Donor** | **Purity** | **Viability** | **Age** | **BMI** | **Cause of Death** | **Studies** | **GSIS Index** |
| --- | --- | --- | --- | --- | --- | --- | --- |
| 1 | 90 | 95 | 56 | 40.1 | Stroke | Basal cAMP | N/A |
| 2 | 95 | 91 | 28 | 24.6 | Head Trauma | Basal cAMP | N/A |
| 3 | 90 | 95 | 39 | 23.7 | Stroke | Basal cAMP, GSIS | 1.11 |
| 4 | 95 | 95 | 44 | 34.6 | Head Trauma | GSIS | 1.33 |
| 5 | 90 | 95 | 38 | 39.8 | Anoxia | Basal cAMP, GSIS | 2.03 |
| 6 | 90 | 95 | 35 | 31.5 | Head Trauma | GS-cAMP, GSIS | 1.4 |
| 7 | 95 | 95 | 51 | 22.5 | Stroke | GS-cAMP | N/A |
| 8 | 90 | 98 | 34 | 49.9 | CNS Tumor | GS-cAMP | N/A |
| 9 | 90 | 90 | 42 | 32.1 | Cerebrovascular  /Stroke | GS-cAMP | N/A |
| 10 | 95 | 95 | 62 | 36.1 | Anoxia | GS-cAMP | N/A |
| 11 | 91 | 95 | 32 | 28.5 | Head trauma | GS-cAMP | N/A |

**S1 Table. Human Islet Donor Characteristics**

**Pratt et al., S1 Table**
