# Supplementary figures and images for: Regulation of cAMP accumulation and activity by distinct phosphodiesterase subtypes in INS-1 cells and human pancreatic β-cells
Source: PLoS One. 2019 Aug 23;14(8):e0215188. doi: 10.1371/journal.pone.0215188 (PMC6707593; doi:10.1371/journal.pone.0215188)

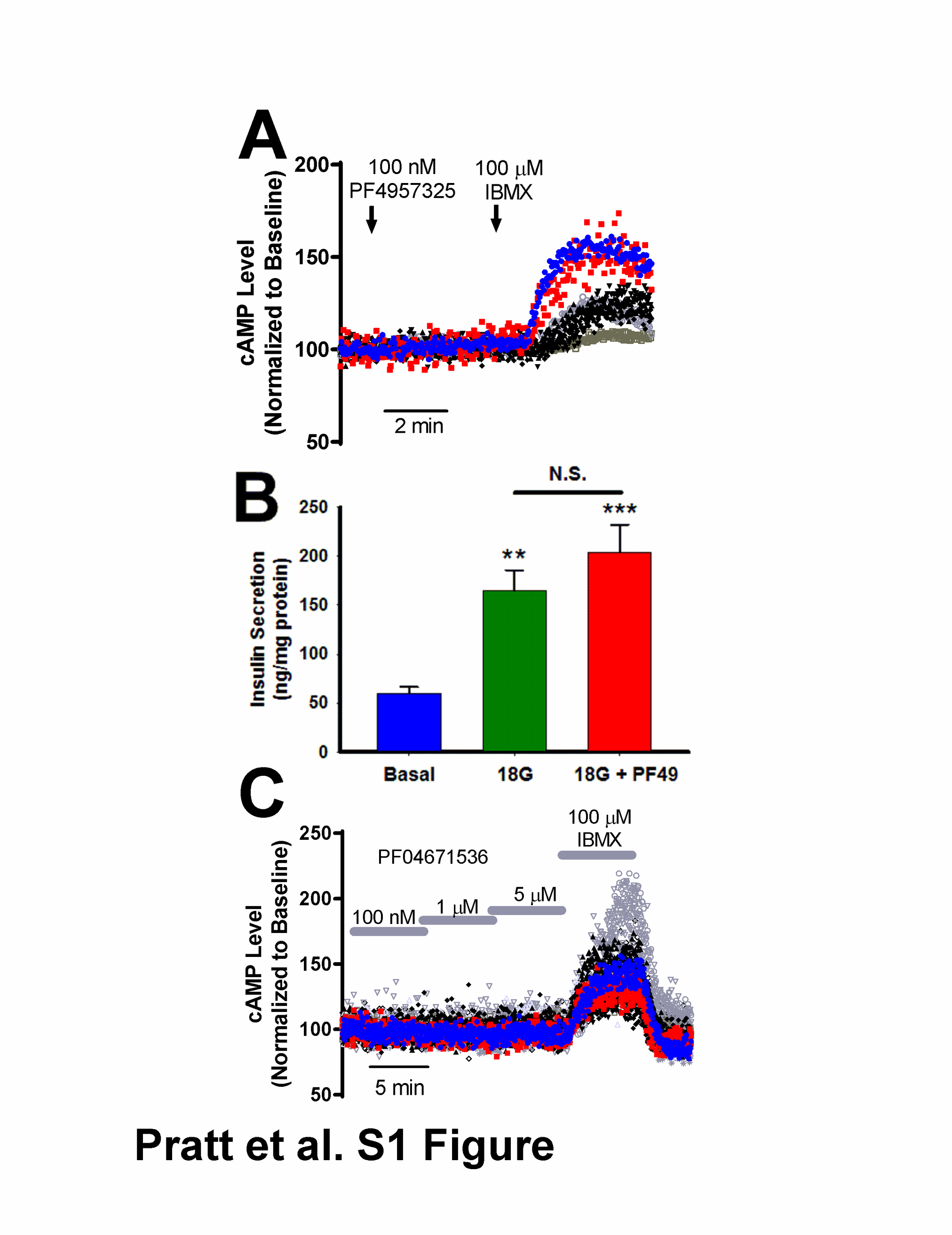

Supplement: S1 Fig — (A) Application of 100 nM PF4957325 on INS-1 in KRBH with 0 glucose resulted in no increase in cAMP over baseline. Subsequent application of 100 μM IBMX resulted in increased cAMP ranging from 7–49% over baseline (n = 14 cells) (B) PF4957325 does not potentiate glucose-stimulated insulin secretion in INS-1 cells. Stimulation of INS-1 cells with 18 mM glucose resulted in a significant increase in insulin secretion over basal secretion. Co-stimulation with 100 nM PF4957325 failed to result in a significant increase in secretion compared to 18 mM glucose alone(n = 3). (C) PF04671536 does not increase intracellular cAMP in INS-1 cells. Application of increasing concentrations of PF04671536 to INS-1 cells in KRBH with 0 glucose failed to increase in cAMP over baseline. Application of 100 μM IBMX resulted in increases in cAMP ranging from 23–113% over baseline (n = 11 cells). (TIF) [file pone.0215188.s002.tif]

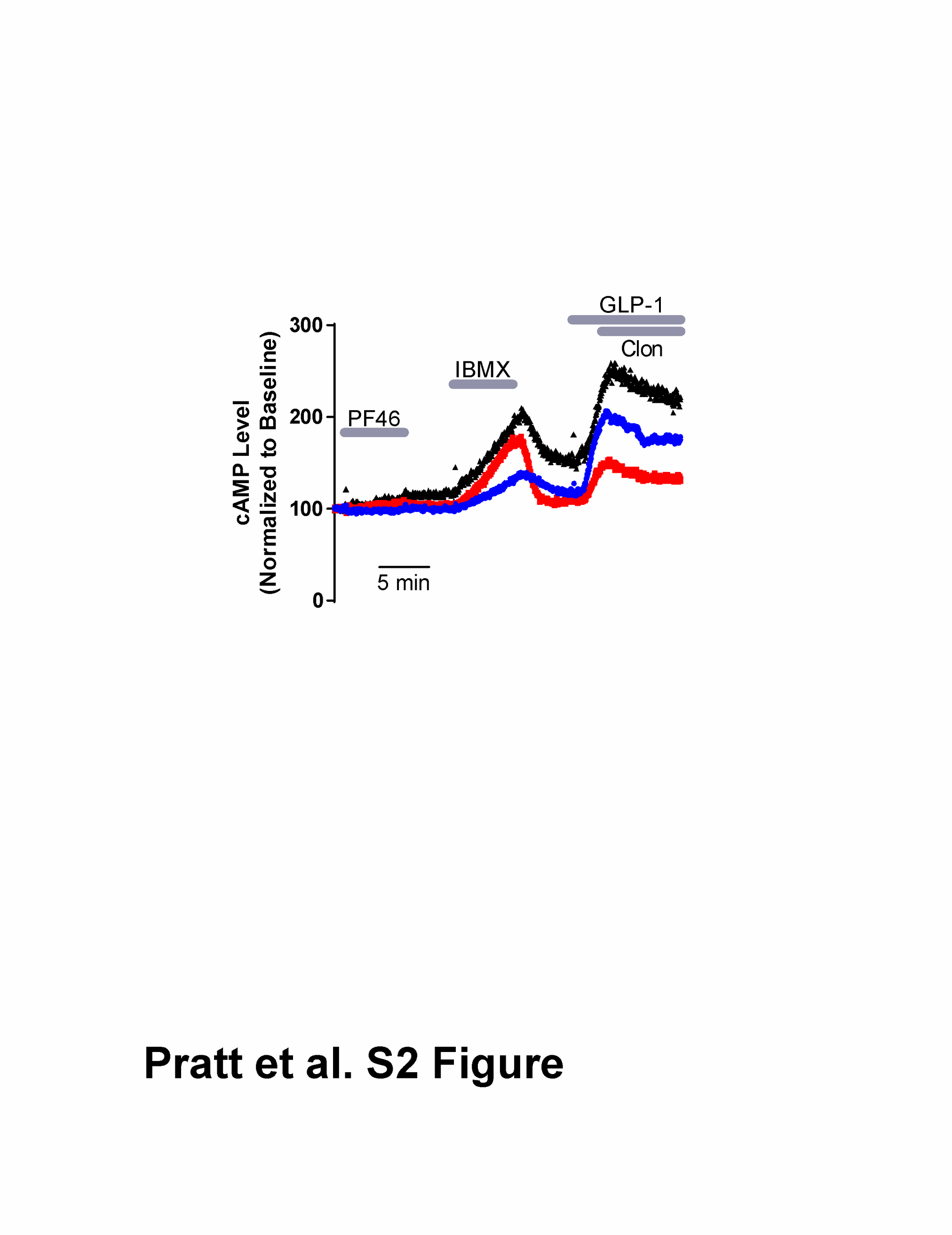

Supplement: S2 Fig — Application of 100 nM PF04671536 to human β-cells in 1.7 mM glucose does not result in any increase in cAMP over baseline. Application of 100 μM IBMX increased cAMP ranging from 40–101%. GLP-1 and clonidine were used to identify β-cells (n = 4). (TIF) [file pone.0215188.s003.tif]
